# The Board of Health—New Public Health Bills

**Published:** 1855

**Authors:** 


### HYGIENIC JURISPRUDENCE.

### THE BOARD OF HEALTH—NEW PUBLIC HEALTH BILLS.

DURING the past quarter, two new Bills—viz., the Nuisances Removal Bill, and the Public Health Bill—have been introduced into the House of Commons by the President of the Board of Health, and are now before a select committee. As these Bills have undergone and are undergoing great modifications, we consider it most prudent to hold back for next number any statement or opinion concerning them, or the objections that have been raised against them. On one point, however, an exceptional remark ought to be made,—viz., that the Bills, as they have at present appeared, seem to have ignored the medical element altogether. This important omission must surely be accidental; for what great step can be made in sanitary legislation without the assistance of the professors of that science which deals exclusively with the issues of health and disease, of life and death?

When the present Board of Health was first established, a medical council was organized, at the suggestion, we believe, of Sir Benjamin Hall, for the purpose of assisting the Board in its labours. This Council, though not satisfactory to the profession in regard to its constitution, was still an important recognition of the value of medical knowledge in relation to hygienic jurisprudence, and we hope that in the revised bills some similar mark of consideration will be supplied.

The Board of Health, during the past quarter, have issued some important papers:—

I. Appendix V. of the Second Report on Quarantine, entitled, "Report on the Plague of Malta in 1818." By Dr. H. Burrell.

II. "Report on Sandgate in connection with the late Visitation of Cholera." By Mr. Blackwall, C. E.

III. "An Explanatory Analysis of the Bill for Consolidating the Nuisance Removal and Diseases Prevention Acts."

IV. "A Report on Epidemic Cholera in the Metropolis in 1854." By Dr. Sutherland. This report is an abstract of the reports of the nine inspectors appointed by the Government,—viz., Drs. Hassall, Greenhow, Mortimer, Glover, Macloughlin, R. King Fraser, and Messrs. Walsh, Glover, and Patterson. The facts recorded in this report are, as might be expected, most valuable. We shall refer to them on another occasion.

V. "A Report on the Common and Modern Lodging-houses of the Metropolis, with reference to the Epidemic Cholera, in 1854." By G. Glover, Esq. In the general summary of this report it is stated that the inmates of the different lodging-houses, which belong to either of the two societies for improving the dwellings of the poor, to private individuals, and those under the surveillance of the police, have enjoyed all but complete exemption from the epidemic.

It is shewn that the Metropolitan Association has lost only five of the inmates of its buildings, or in the proportion of 34 in 10,000; but if four fatal cases which occurred in the Albert Street buildings for families, having originated from other causes, be excluded, the mortality would only have been in the proportion of 7 in 10,000.

In the Society's buildings for improving the condition of the labouring classes, there has been only one death, and that can scarcely be said to have originated in them; and if all the deaths in the buildings of the two societies were taken together, the mortality would only be in the ratio of 26 in 10,000.

Whilst the working of the Common Lodging-Houses' Acts has been attended with the best possible results, and has far exceeded the anticipation of their promoters, the conclusions to be drawn from these results are seriously affected by the migratory character of the population, and other causes.

Again, if either the societies' buildings or the common lodging-houses are compared with certain other portions of the metropolis, or with the metropolis as a whole, it is evident that a very great improvement in the health of the inhabitants of these tenements has been effected. Thus, in "the Potteries," Kensington, which still retains its unenviable character for insalubrity, the mortality from cholera and its adjuncts in this place has been at the rate of 259 to 10,000, whilst in all the society's buildings together it has not been above 27 to 10,000. The mortality, therefore, has been nine times greater in "the Potteries" than in the model lodging-houses.

The facts adduced appear to prove, that in proportion as we improve the dwellings of the people, and secure them proper ventilation, good drainage, sufficient supply of water, and inoffensive water-closet accommodation, we advance the standard of their general health, and exempt them from cholera and other epidemic diseases.

